# Nonaqueous Oxidation in DNA Microarray Synthesis Improves the Oligonucleotide Quality and Preserves Surface Integrity on Gold and Indium Tin Oxide Substrates

**DOI:** 10.1021/acs.analchem.3c04166

**Published:** 2024-01-29

**Authors:** Erika Schaudy, Gisela Ibañez-Redín, Etkin Parlar, Mark M. Somoza, Jory Lietard

**Affiliations:** † Institute of Inorganic Chemistry, University of Vienna, Josef-Holaubek-Platz 2, Vienna 1090, Austria; ‡ Leibniz-Institute for Food Systems Biology at the Technical University of Munich, Lise-Meitner-Straße 30, Freising 85354, Germany; § Chair of Food Chemistry and Molecular Sensory Science, Technical University of Munich, Lise-Meitner-Straße 34, Freising 85354, Germany

## Abstract

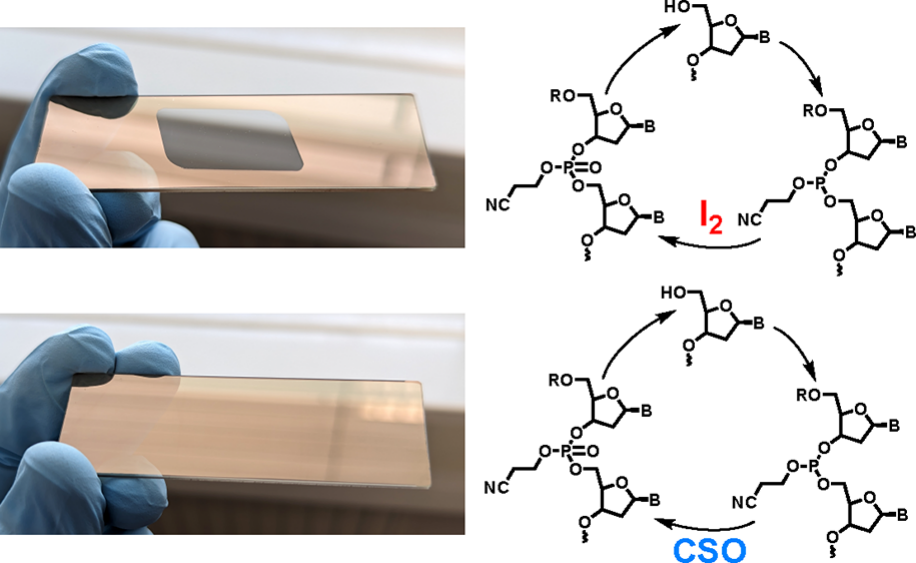

Nucleic acids attached to electrically conductive surfaces are very frequently used platforms for sensing and analyte detection as well as for imaging. Synthesizing DNA on these uncommon substrates and preserving the conductive layer is challenging as this coating tends to be damaged by the repeated use of iodine and water, which is the standard oxidizing medium following phosphoramidite coupling. Here, we thoroughly investigate the use of camphorsulfonyl oxaziridine (CSO), a nonaqueous alternative to I_2_/H_2_O, for the synthesis of DNA microarrays in situ. We find that CSO performs equally well in producing high hybridization signals on glass microscope slides, and CSO also protects the conductive layer on gold and indium tin oxide (ITO)-coated slides. DNA synthesis on conductive substrates with CSO oxidation yields microarrays of quality approaching that of conventional glass with intact physicochemical properties.

## Introduction

Oligonucleotide synthesis and phosphoramidite chemistry are so intertwined that they can be considered synonyms despite recent colossal efforts,^1−4^ aiming at dethroning this (by all chemical accounts) ancient technology.^5^ Phosphoramidite chemistry has so far prevailed because of its extremely high nucleotide coupling yield, versatility, reliability, and fairly simple chemistry of nucleic acid assembly.^6^ Robustness notwithstanding, the coupling chemistry has been the subject of optimization and adaptation in order to increase the yield and efficiency to accommodate DNA, RNA, and chemical modifications and to allow a high-throughput synthesis on microarrays.^7−9^ A central aspect of nucleotide coupling is the formation of the internucleotidic phosphodiester bond that usually proceeds through the oxidation of a P(III) phosphite triester into a P(V) phosphotriester and is most commonly mediated by iodine and water. The mixture is inexpensive, and the oxidation reaction is complete in mere seconds. However, there are select cases where the iodine/water cocktail is undesirable. For instance, methylphosphonite functions are significantly more prone to hydrolysis than phosphite triesters and so require low-water oxidizers.^10−12^ Other carbon-bearing phosphorus species like phosphonoacetate (PACE) likely suffer from the same sensitivity to water and therefore require a change in the oxidation protocol.^13^ The repeated use of I_2_/H_2_O oxidizers also puts oxidizable nucleobases at risk of unwanted base oxidation or oxidative cleavage, most commonly guanine and guanine derivatives.^14^ Chemical modifications containing functional groups that are sensitive to oxidative conditions require oligonucleotide synthesis to be carried out with nonaqueous oxidizers.^15,16^ The water content of standard oxidizing solutions (typically 10% in THF/pyridine) adds to the necessity for thorough washing of the solid support in order to avoid water contamination during the subsequent coupling stage. Iodine itself can participate in undesired side reactions and can lead to phosphotriester bond cleavage when using sterically hindered tertiary phosphoramidites.^17,18^ Likewise, phosphorothioate di- and triesters are sensitive to strand cleavage, much more so than phosphodiesters,^19^ by a process of alkylation at the negatively charged sulfur.^20^ In the presence of iodine, a charged S^–^ can produce a strong S–I bond that can further lead to strand cleavage or sulfur/oxygen exchange.^21^ Beyond the chemistry of oligonucleotide elongation, the surface on which nucleic acid synthesis takes place can be affected by the use of conventional oxidizers. For the in situ synthesis of nucleic acid microarrays, where the final oligonucleotide product is expected to remain bound to its substrate, the stability of the surface is of paramount importance. With DNA microarray synthesis exploring new avenues away from standard glass and toward conductive surfaces such as indium tin oxide (ITO),^22^ polymer and carbon-based substrates,^23,24^ or silicon wafers,^25^ ensuring the integrity of the microarray substrate and maintaining equal efficiency to synthesize on silanized slides^26,62^ is crucial. Metal-coated microscope slides risk being oxidized by the action of iodine and water and thus produce a water-soluble compound^27^ or otherwise change the physicochemical properties of the coating.^28^ Alternatives to aqueous oxidizers for DNA synthesis exist.^29^ Peroxides have been used with good results,^30−32^ but the innate fragility of peroxides encourages the use of alternatives. Oxaziridines readily oxidize P(III) compounds,^33^ and the camphorsulfonyl version (CSO) has been successfully applied to oligonucleotide synthesis requiring particular attention to the oxidizing conditions.^13,18,34−38^ A solution of CSO in acetonitrile is commercially available and can be readily diluted. In the context of our expanding chemical toolbox for the synthesis of modified oligonucleotides on microarrays^39−41^ and our desire to adapt the protocols for microarray photolithography to non-traditional substrates, we explore in this study the synthesis parameters allowing for efficient and reliable CSO-mediated oxidation of oligonucleotides synthesized in situ on glass and on diverse metal-coated substrates. We found that DNA synthesis with stepwise CSO oxidation is as effective as I_2_-mediated oxidation, producing libraries of high quality, and, importantly, that it is efficient and compatible with gold and ITO-coated microscope slides.

## Experimental Section

### Reagents and Materials for Oligonucleotide Microarray Synthesis

Photolabile DNA phosphoramidites (5′-BzNPPOC^42^) were purchased from Orgentis, and dry acetonitrile (<30 ppm of H_2_O) was from Sigma-Aldrich (L010000) along with molecular trap packs (Z509000 and Z509027) that remove moisture from the dissolved phosphoramidites (0.03 M in ACN). A 1% (*w*/*v*) solution of imidazole in DMSO (Sigma, 56750 and 276855) serves as the exposure solvent during 5′-BzNPPOC photodeprotection, which is carried out using 365 nm UV light produced by a high-power UV-LED (Nichia NVSU333A). Activators were either 0.25 M dicyanoimidazole in ACN (Sigma L032080) or 0.25 M 5-(ethylthio)-1*H*-tetrazole in ACN (Sigma L003080). A base-sensitive dT monomer, used to cleave oligonucleotide libraries from the surface of the array,^43^ equipped with either a 5′-DMTr or a 5′-NPPOC was purchased from ChemGenes. DMTr removal was effected with 3% trichloroacetic acid in dichloromethane (Sigma L020000). Oxidizers were either 0.02 M I_2_ in tetrahydrofuran/pyridine/H_2_O 90:9:0.4 (Sigma L060020) or 0.5 M CSO in ACN (Eurogentec 40–4632–52E). Microarray synthesis was carried out on glass microscope slides (Schott Nexterion D) silane-functionalized with *N*-(3-triethoxysilylpropyl)-4-hydroxybutyramide (Gelest SIT8189.5), gold-coated microscope slides with a 3D-amine layer (PolyAn 109 10008), or ITO-coated SuperEpoxy 2 microscope slides (ArrayIt). Deprotection was performed with a 1:1 mixture of ethylenediamine (EDA, Sigma 03550) in ethanol (VWR 1009831000), and the cleavage and deprotection of oligonucleotide libraries were alternatively carried out in a 1:1 solution of EDA in toluene (Sigma 244511).

### Principle of Microarray Photolithography

The process of nucleic acid microarray synthesis by maskless photolithography (MAS^44^) has been extensively described elsewhere.^41,45−51^ For the sake of brevity, only the essential aspects of the process will be discussed here. Oligonucleotide elongation follows the same principle as conventional solid-phase synthesis, meaning that synthesis proceeds through the stepwise addition of nucleotides in a cycle-based approach with repeating chemical reactions following the order 5′-deblocking → coupling → oxidation. The delivery of reagents and phosphoramidites is accomplished by an automated DNA synthesizer (Expedite 8909, PerSeptive Biosystems). The reaction cell contains two functionalized glass slides mounted over a quartz block^52^ and is precisely positioned at the focal plane of incoming UV light. At the UV illumination stage, patterned UV light exposes the two slides simultaneously with the 50 μm-thick inner chamber between the slides immersed in the exposure solvent (1% imidazole in DMSO). Patterned UV light is produced by reflection over a 0.7 in. digital micromirror device (DMD, Texas Instruments) where micromirrors have been turned ON or OFF, with ON mirrors exposing the corresponding area on the surface of the slide. The ON and OFF selection occurs by loading a bitmap file (digital mask) containing white (ON) and black (OFF) pixels into the DMD. Digital masks are generated by a custom-built script on MATLAB (Mathworks). UV-mediated photodeprotection is carried out at a radiant exposure of 3 J/cm^2^ or 6 J/cm^2^ for off-array applications. Gold and ITO slides are not fully transparent (20 and 63% light transmittivity, respectively), so to properly expose the second lower glass slide, the UV illumination time was adjusted to take the low transparency into consideration (to 212 and 66 s, respectively, vs 42 s for 3 J/cm^2^ radiant exposure on standard glass). The phosphoramidite coupling reaction is considered complete at >99% stepwise efficiency after 15 s. After coupling, the synthesis area is dried by passing a stream of helium gas, setting the stage for the oxidation reaction.

### In-Cell and Off-Cell Oxidation Tests

In routine experiments, oxidation is performed during synthesis and at each cycle in the reaction cell (“in-cell”). Iodine/water-mediated oxidation was performed for 3 s with the oxidation solution flowing through the reaction chamber in seven consecutive pulses of ∼16 μL each. Each pulse has a delivery rate of ∼25–30 ms, totaling ∼3 s of delivery and contact time between the substrate and the oxidation solution. Iodine-mediated oxidation is followed by an ACN wash. For CSO-based oxidation, the reaction time was routinely set at 20 s stepwise followed by a final 3 min. In post-synthesis P(III) oxidation (“off -cell”), a four-tiled square-shaped hybridization chamber (Grace Biolabs, custom cut) is placed atop the synthesis area. The synthesis area was divided into four parts that can be separately addressed by any of the four subchambers. On one microarray, the subchambers are filled with 60 μL of the iodine oxidizer and left for 10, 30, 1, or 5 min. A second microarray has the applied hybridization chamber filled with 0.5 M CSO and left for 30 s, 1, 3, or 5 min. In all cases, the oxidizing solution was pipetted out, and the corresponding subchamber was washed thoroughly with ACN (4 × ). The hybridization chamber was then peeled off, and the slide was dried in a microarray centrifuge and subjected to DNA deprotection as described in the next section.

### Microarray Deprotection, Hybridization, and Data Extraction

After synthesis, the nucleobase and phosphodiester protecting groups are removed by immersing the slide in the EDA/EtOH solution for 2 h at rt followed by rinsing with deionized water and drying in a microarray centrifuge. The arrays were then hybridized in a hybridization chamber (Grace Biolabs) with a mixture consisting of 150 μL of 2× MES hybridization buffer, 110 μL of nuclease-free water, 13.3 μL of acetylated BSA (10 mg/mL), and 26.7 μL of the 100 nM Cy3-labeled complementary strand at 42 °C for 2 h in a hybridization oven (Boekel Scientific). After hybridization, the chamber was stripped off, and the array was washed for 2 min in non-stringent wash buffer (SSPE; 0.9 M NaCl, 0.06 M phosphate, 6 mM EDTA, 0.01% Tween20), then 1 min in stringent wash buffer (100 mM MES, 0.1 M NaCl, 0.01% Tween20) followed by a wash in final wash buffer (0.1× SSC) for a few seconds. The arrays were dried in a microarray centrifuge and then scanned on a GenePix 4100A microarray scanner (Molecular Devices) at 5 μm resolution with an excitation wavelength of 532 nm. Data extraction of the scanned images was performed with NimbleScan 2.1 (NimbleGen), and analysis was performed with Microsoft Excel.

## Results and Discussion

### Evaluating the Performance of CSO Oxidation on DNA Microarrays

**Figure 1:**
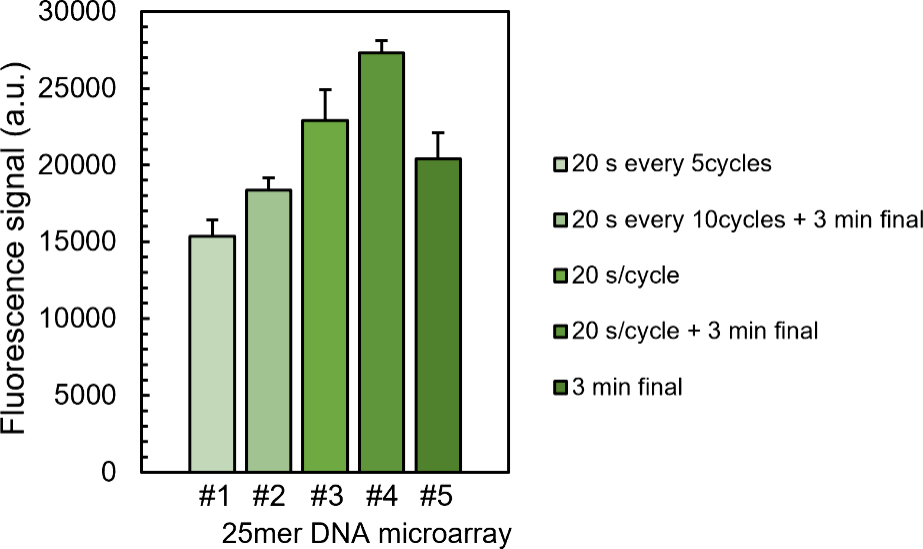
Fluorescence hybridization signals (au) to a 25mer DNA sequence synthesized by microarray photolithography under various CSO (0.5 M in ACN) oxidation conditions. Cycles are phosphoramidite coupling cycles. Error bars are the SD.

The initial tests we performed with 0.5 M CSO oxidation aimed at evaluating its efficiency at various reaction times. To do so, we grow a 25-mer DNA oligonucleotide on functionalized glass slides. After deprotection, we proceeded with a hybridization step. The fluorescence signal of hybridization to a Cy3-labeled complementary strand is an excellent indicator of synthesis and oxidation efficiency as any unoxidized P(III) bond will be degraded during deprotection, leading to DNA loss. We tested various reaction times with intermittent or stepwise addition of CSO. The fact that oxidation can be performed at any given time point during microarray synthesis is a direct consequence of the absence of an acidic deblocking step in photolithography. The stability of the P(III) phosphite triester is therefore limited only by the acidity of the activator. We routinely use dicyanoimidazole (DCI) as an activator,^46^ which has one of the highest p*K*_a_ values among conventional activators, allowing for P(III) bonds to survive for an extended amount of time. Hybridization signals show that intermittent CSO oxidation (every 5 or 10 coupling cycles, arrays #1 and #2; Figure 1) is not as effective as oxidation immediately after coupling (#3). This would indicate either partial P(III) degradation in between contact with CSO as a result of activator acidity or P(III) degradation during DNA deprotection as a result of incomplete CSO oxidation. A 20 s stepwise oxidation time is sufficient to yield very high signals, which are above that of a single final 3 min-long oxidation reaction (#5). Combining stepwise and final oxidation yields the strongest hybridization signal (#4), and this approach was selected as the optimal CSO-mediated conditions for P(III) → P(V) oxidation.

**Figure 2:**
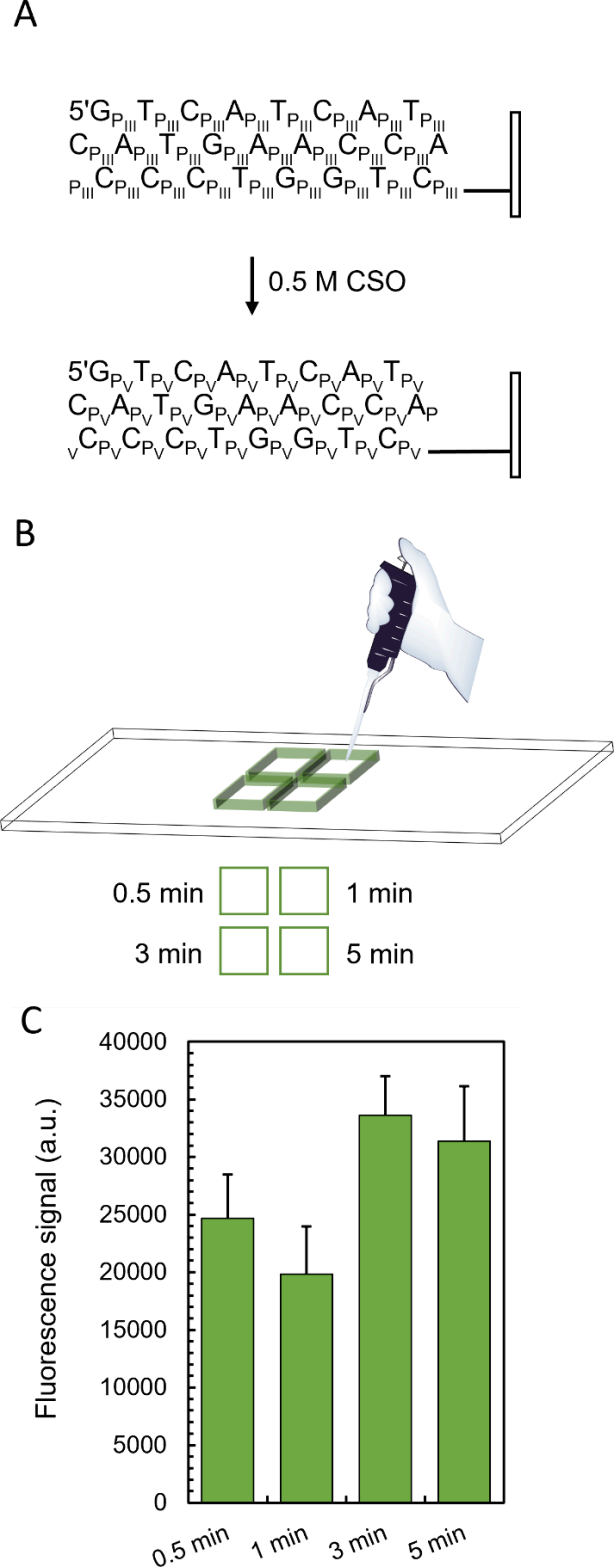
Post-synthetic oxidation of DNA microarrays with CSO. (A) The single final oxidation reaction was carried out as the last step in 3’ → 5’ photolithographic synthesis of a 25mer DNA microarray. (B) Schematic representation of the oxidation reaction performed outside of the photolithographic reaction chamber (“off-cell”) using a self-adhesive, tile-shaped hybridization chamber filled with 0.5 M CSO solution and left to react with the surface-bound DNA for the specified amount of time (0.5, 1, 3, or 5 min). (C) Fluorescence hybridization signals (au) recorded on the same microarray after P(III) oxidation under various CSO reaction times and subsequent DNA deprotection. Error bars are the SD.

We also looked at the kinetics of the single CSO-mediated oxidation reaction carried out post-synthesis (Figure 2A). We performed post-synthesis CSO oxidation under a range of reaction times between 30 s and 5 min on the same microarray (Figure 2B). While 30 s and 1 min of contact with CSO solution already provide satisfactory hybridization signals to the Cy3-labeled DNA complement, the complete P(III) transformation to P(V) appears incomplete since 3 and 5 min oxidation times with CSO yield significantly greater fluorescence values (Figure 2C). The 1 min data point gives out a lower signal than the 30 s data point, which is likely a consequence of a generally lower synthesis quality in the top right corner of the synthesis area.^27^

**Figure 3:**
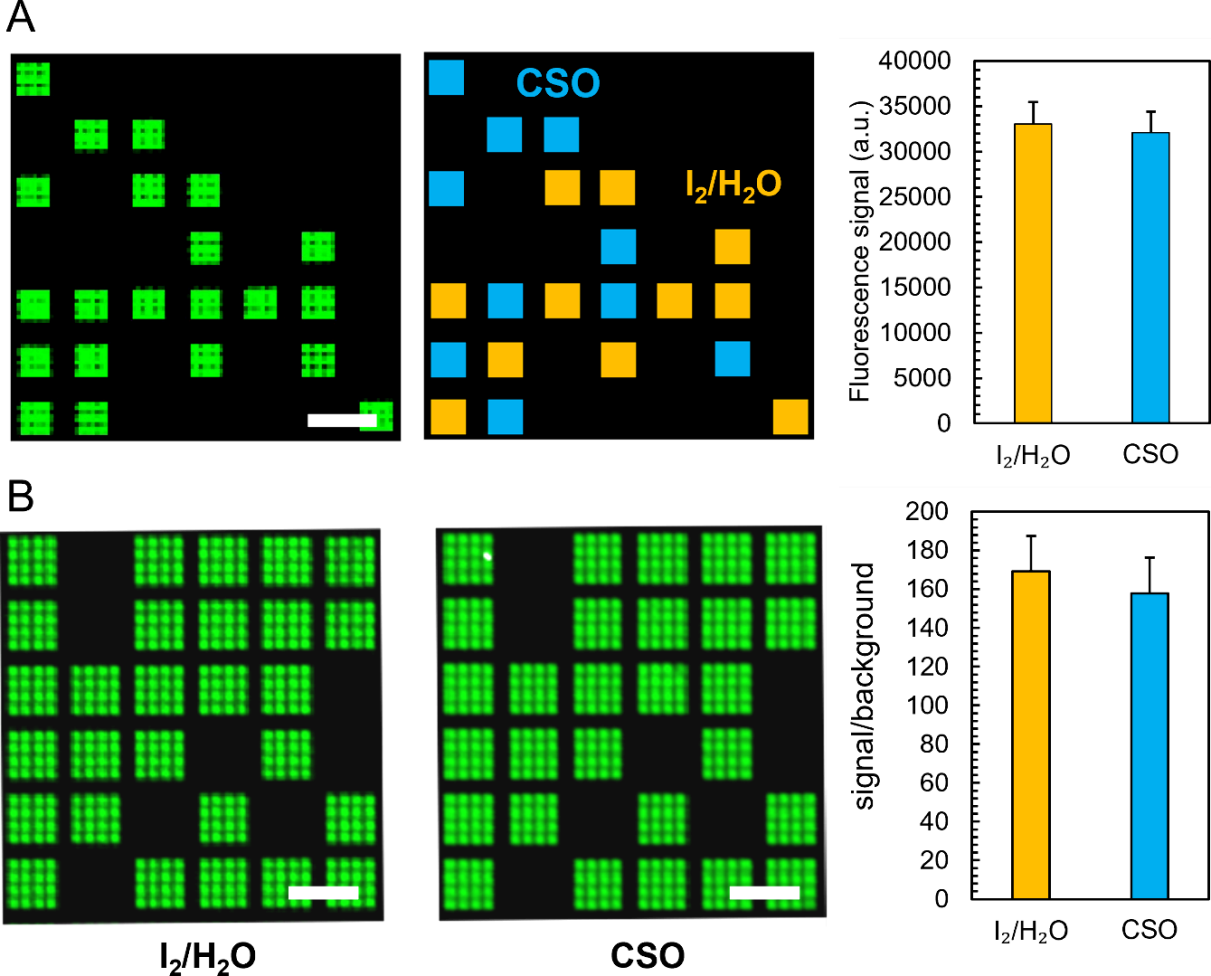
Comparative study of iodine and CSO-oxidized DNA microarrays using hybridization with a fluorescently labeled complementary strand. (A) 25mer DNA oligonucleotides synthesized in series on a single microarray. The 25mer with stepwise iodine-mediated P(III) oxidation is carried out first (features colored in yellow), and the 25mer with stepwise CSO oxidation (20 s) with a final 3 min-long oxidation is carried out next (colored in blue). Each feature is a matrix of 3 × 3 mirrors, and all features are randomly distributed across the surface of the array. On the left is a scan excerpt, and on the right is an illustration of the distribution of features that received either iodine or CSO as an oxidizer. (B) Hybridization to 25mer DNA oligonucleotide microarrays carried out either with iodine- or CSO-mediated oxidation. Each feature is a matrix of 5 × 5 mirrors, and all features are randomly distributed across the surface of the array. Signal/background ratios are calculated as the ratio between feature fluorescence and background fluorescence (black, non-synthesized features). Scan excerpts represent <0.5% of the total synthesis area. Scale bar is ∼100 μm. Errors bars are the SD.

We next wanted to compare oxidation efficiencies between CSO and standard I_2_/H_2_O. While the photochemistry of microarray photolithography is spatially selective, the oxidation reaction is not and equally affects all P(III) bonds across the surface of the array. To ensure a fair comparison between the two oxidation methods, we therefore synthesized two 25mers in series, each with a unique oxidation procedure: one with conventional iodine-mediated oxidation (3 s stepwise) followed by one with CSO oxidation (20 s stepwise followed by a final 3 min reaction). Hybridization results reveal equal fluorescence signals for the I_2_ and CSO-oxidized 25mers (Figure 3A), indicating comparable oxidation efficiencies and a fluorescence intensity homogeneously distributed across the surface. For microarray surfaces subjected solely to CSO oxidation, the background fluorescence remains very low (<100 au) and within the same range as that for I_2_-treated DNA arrays, meaning similar signal/background ratios, in the order of 160:1 (Figure 3B). These results suggest that replacing an aqueous oxidizer with an organic alternative does not noticeably affect the surface properties of silanized glass slides.

**Figure 4:**
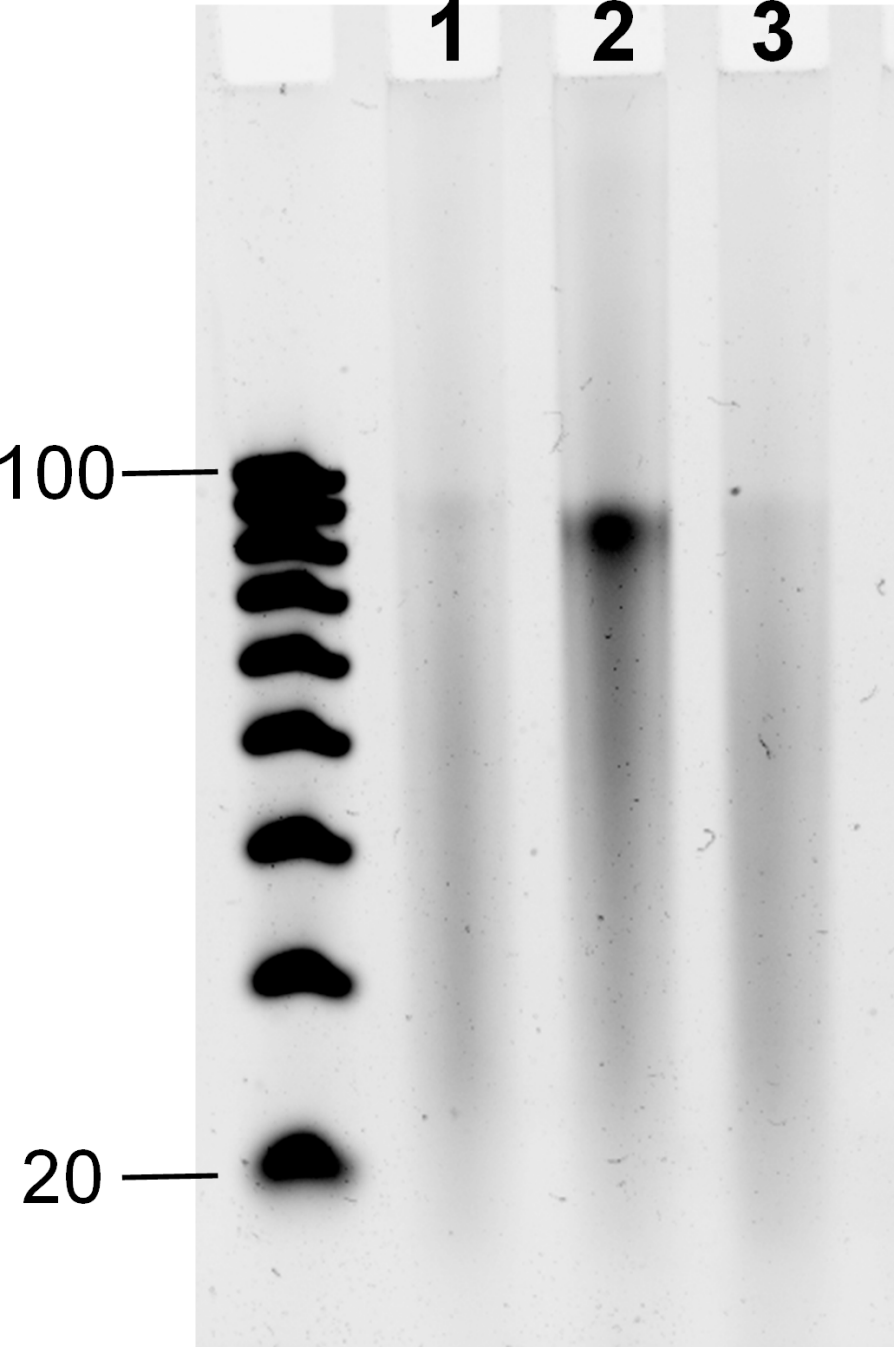
Denaturing 20% polyacrylamide gel electrophoresis of crude 97mers synthesized by MAS. The oxidation protocol was either I_2_/H_2_O (3 s stepwise, lane 1, or 30 s stepwise, lane 3) or 0.5 M CSO (20 s stepwise, lane 2). The oligonucleotides were synthesized with BzNPPOC DNA phosphoramidites using ETT as the activator and 6 J/cm^2^ of radiant exposure. Amount loaded for each sample was 100 ng. Left: DNA ladder (20–100 nt). The uncropped gel is available in Figure S1.

We then applied the CSO oxidation procedure to the synthesis of longer oligonucleotides, aiming at analyzing the synthesis quality by recovering DNA from the surface of the array. This method for the preparation of nucleic acid libraries requires the initial incorporation of a base-sensitive monomer to conveniently cleave the DNA during deprotection.^26,53,54^ For off-array applications, we typically employ 5-ethylthiotetrazole (ETT) as an activator since the hybridization signal homogeneity, which is severely disturbed on microarrays synthesized with tetrazole derivatives as an activator,^46^ is no longer a concern for cleaved nucleic acid libraries. We synthesized a single 97-nt long DNA oligomer across the entire synthesis area under three different conditions, only changing the oxidation protocol: either a short (3 s) or long (30 s) stepwise iodine-mediated oxidation or a stepwise CSO approach. After cleavage and quantification, the DNA samples were analyzed by gel electrophoresis (Figure 4). The CSO-oxidized DNA crude is considerably cleaner than the iodine-/water-oxidized versions, displaying a clear band at full-length and fewer impurities when the full-length material in iodine-oxidized DNA is much fainter. In the CSO variant, the majority of the crude DNA elutes at around the 90-nt mark. This would suggest an ∼90% stepwise cycle efficiency. However, salt impurities are likely to be found in large amounts in the sample due to the use of ethylenediamine as the cleavage medium, which remains as an organic layer that dissolves upon the addition of water. The resulting cationic ethylene diammonium can certainly affect the mobility during electrophoresis.

### Surface Properties of Coated Microscope Slides with CSO Oxidation

In detection and biosensing applications, surfaces decorated with nucleic acids often need to accommodate a functional layer between substrate and DNA usually in the form of an electrically conductive material. We have previously worked on the photolithographic synthesis of DNA microarrays on ITO-coated microscope slides^22,55^ but found that the fluorescence hybridization signal on ITO arrays was always markedly lower than that of conventional silanized glass. While this effect may be the result of fluorescence quenching, we also questioned whether the synthesis efficiency on ITO is much below that of glass. We suspected that the iodine and water combination can react with the inorganic layer, washing it off along with the synthesized DNA during microarray fabrication. This effect appeared to be immediately obvious when attempting to prepare DNA microarrays on gold-coated substrates (Figure S2). Repeated use of the iodine/water mixture to oxidize P(III) bonds after each coupling resulted in a completely eroded gold layer, leaving behind a transparent area that corresponds to the cross-section of the reaction chamber. Alternative oxidation approaches to safeguard the metal coating, for instance, with a very short (3 s) post-synthetic iodine-mediated oxidation, did not visibly damage the surface but at the cost of a fairly low hybridization signal (vide infra). A longer final oxidation time (5 min) with iodine/water already deteriorated the gold layer. We also considered hydrogen peroxide as an alternative to iodine. We found that a single final oxidation reaction carried out for 10 min with a freshly prepared solution of 0.25 M H_2_O_2_ in ACN kept the substrate intact, but, again, the synthesis yield was poor, indicating a low oxidation efficiency. Conversely, stepwise CSO-mediated oxidation had no visible effect on the metal coating of gold microscope slides and, as discussed further below, noticeably improved hybridization signals as well. In comparison, the ITO-coated microscope slides fared better in terms of surface protection and were kept in good condition in both iodine and CSO-mediated oxidation routes. The purple-tinted layer of ITO is clearly visible in both cases with no obvious coating discontinuity at the DNA synthesis site (Figure S2).

**Figure 5:**
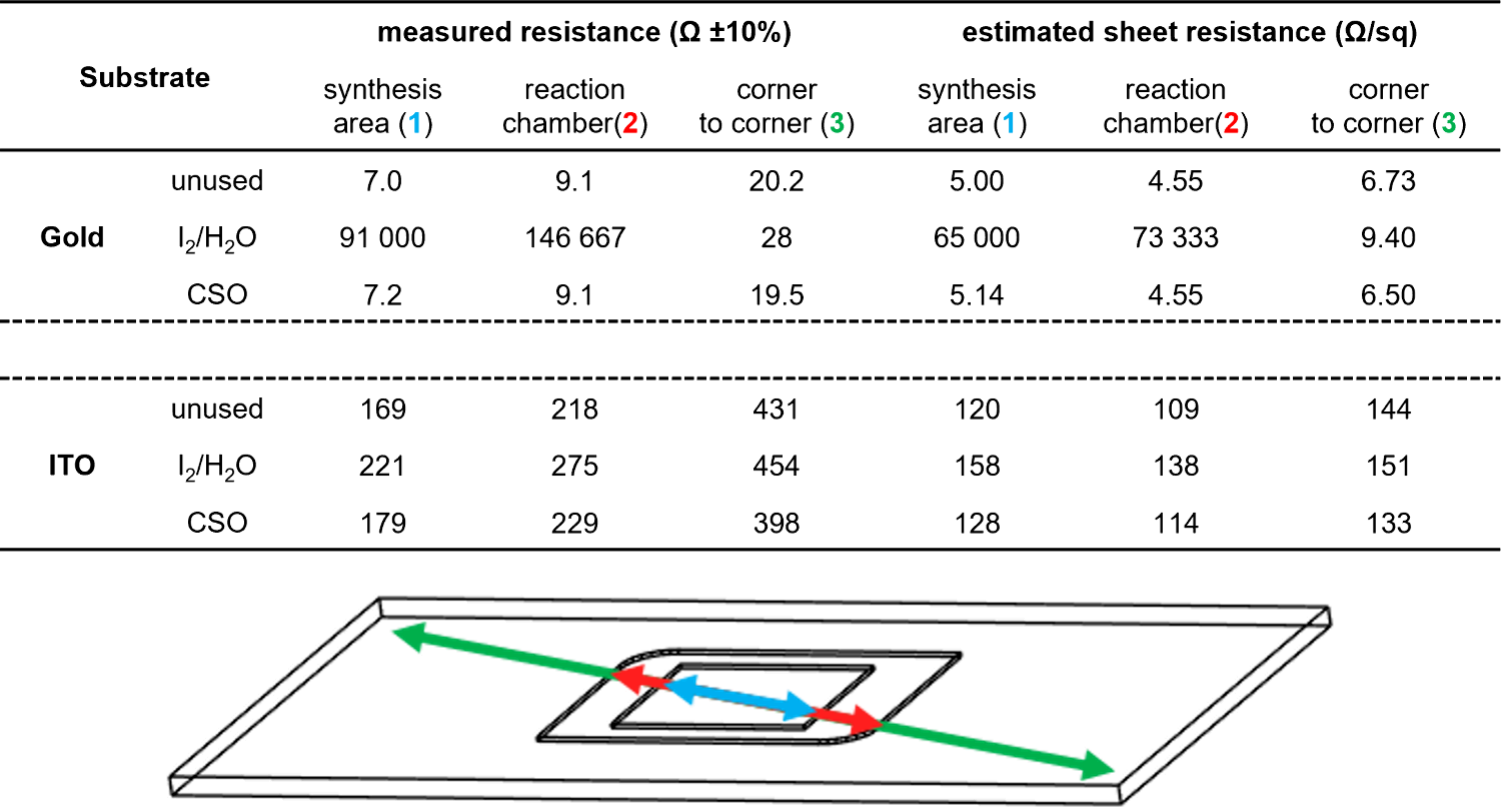
(top) Resistance of gold- and ITO-coated microscope slides before and after DNA synthesis under iodine or CSO-mediated oxidation conditions. Measurements were taken with an ohmmeter at three diagonally opposed locations (bottom): slide corners (green), within the reaction chamber (red), and within the MAS synthesis area (blue). Sheet resistance (in Ω/square) was estimated by dividing the resistance with the length/width ratio of the rectangle encasing the two measurement points. The ratio was ∼3:1 for the slide dimensions, 2:1 for the reaction chamber, and 1.4:1 for the synthesis area. The sheet resistance and electrical resistance should be understood as approximate values only with an error margin of at least ±10% across three independent measurements.

We also monitored the conductive properties of both gold and ITO slides after they were subjected to photolithographic DNA synthesis with iodine or CSO as the oxidation medium. To do so, we measured electrical resistance using a simple ohmmeter between two points at three different diagonal corners: the slide itself, at the zone of contact with synthesis reagents, and on the synthesis area proper (Figure 5). Sheet resistance, which is commonly employed to characterize surface conductivity, was estimated based on the length/width ratio of the rectangular strip bound by the two electrodes. In doing so, the sheet resistance is expected to be invariable across all three measurements. Unsurprisingly, electrical resistance for the gold-coated slide has been heavily affected by DNA synthesis with iodine, and the eroded section logically shows extremely high resistance values of close to 100 kΩ when the same section on an unused slide was rated at 7–9 Ω. Only between diagonally opposing corners of the slide can electrical conductance still be measured as a conductive path can still be traced across the remaining gold-coated segment. The DNA microarray synthesized on a gold-coated slide with CSO shows little to no change in electrical resistance after synthesis, suggesting that repeated contact with iodine and water is the main contributor in solubilizing gold and other reagents and solvents are not participating. With virtually no change in the sheet resistance either, CSO-oxidized DNA microarrays appear to leave the gold layer intact at both the macroscopic and microscopic levels. The ITO-coated slide also appears to have been affected by contact with the I_2_/H_2_O oxidizer despite visual inspection revealing no noticeable change. At both the synthesis area and the reaction chamber, the electrical resistance has increased by almost 30%. With the CSO-oxidized ITO slide, resistance values at the synthesis area and reaction chamber can be considered to be almost identical to the unused version, meaning that overall, in both gold- and ITO-coated slides, CSO is the superior oxidation medium that guarantees surface integrity.

**Figure 6:**
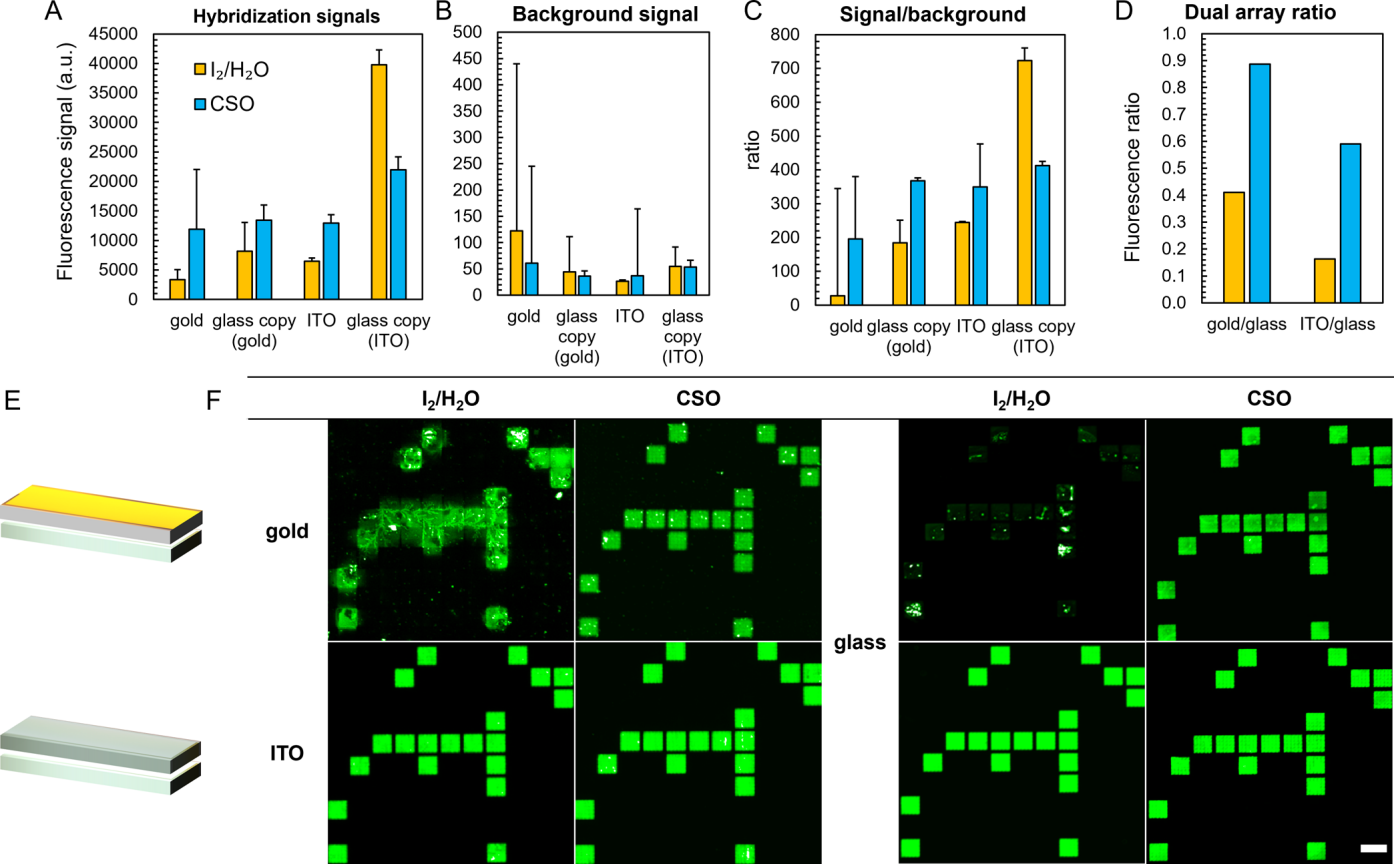
(A) Fluorescence signals with a Cy3-labeled complementary sequence hybridized onto 25mer DNA microarrays decorated with ∼2000 replicates of the same sequence. Synthesis was performed using either iodine/water (yellow bars) or CSO (blue bars) as the oxidizer and otherwise standard photolithographic conditions on gold- or ITO-coated microscope slides. The glass copy (gold/ITO) labeling refers to the signal from the glass slide when synthesized alongside a gold- or ITO-coated substrate. (B) The background signal is recorded from features that only contain a dT_5_ linker. (C) The signal/background corresponds to the ratio between hybridization signal and background fluorescence. (D) The dual array ratio is the ratio between fluorescence signals on the conductive substrate and those of the glass microscope slide. Error bars are the SD. (E) In the dual-array MAS procedure, a second microarray is produced simultaneously on a common silanized glass slide (“glass copy”). (F) Excerpts of microarray scans after hybridization with a Cy3-labeled complementary 25mer DNA strand. Hybridization was performed on gold and ITO-coated microscope slides and their duplicate on silanized glass. Each feature is a matrix of 5 × 5 micromirrors with a one-mirror wide gap between adjacent features. Features are randomly distributed across the entire microarray, and all contain the same 25mer DNA sequence. Excerpts are ∼0.5% of the total synthesis area. The scale bar is ∼100 μm.

To question whether an undamaged surface also correlates with greater synthesis efficiency, we recorded hybridization signals on gold and ITO slides and compared the results to the signals recorded on silanized glass. Our system outputs two microarrays per run on two separate slides,^52^ allowing for direct comparison between standard glass and conductive substrates (Figure 6D). Legacy iodine-mediated DNA synthesis leads to very poor hybridization signals with a 25mer oligonucleotide on the damaged gold substrate in the order of a few thousand units where signals upward of 20,000 units are commonplace (Figure 6A). Gold substrates that were only treated once for 3 s post-synthesis with an iodine oxidizer, while not detrimental to surface integrity, led to signal intensity not exceeding ∼2000 au, which is similar to terminal oxidation with H_2_O_2_ (Figure S3). Examination of the high-resolution scan on the damaged gold slide reveals a heavily distorted fluorescence distribution with a very intense intra-feature signal inhomogeneity and large swaths of the scanned area either poorly resolved or affected by blur and signal creep outside of the intended feature (Figure 6F). The background itself is noticeably high (>100 au;Figure 6B) with bright fluorescent spots dotting the surface, which is a fairly uncommon occurrence on otherwise standard microarrays. The duplicate DNA microarray on the corresponding glass slide appears to have been affected by the poor synthesis results on the gold substrate with a modest fluorescence intensity (<10,000 au) mostly stemming from saturated signals randomly dotting the surface (Figure 6F). The reason why synthesis on glass is equally inefficient in this scenario is not entirely clear, but the gold layer being gradually solubilized during iodine oxidation may not be fully washed away before proceeding with the UV exposure step, which would likely affect the photolysis efficiency. Replacing the iodine for the CSO oxidizer has significantly improved the hybridization efficiency with signals four times higher than for the I_2_/gold setup and much cleaner, fully resolved scans (Figure 6F). The background noise is also more uniform. Some bright fluorescent spots remain visible particularly within the DNA features themselves, indicating that CSO exchange has not resolved all issues associated with synthesis on gold surfaces. The duplicate glass is brighter with fluorescence being more homogeneously distributed across each feature. The ratio between hybridization signals on the CSO-oxidized gold substrate and its glass duplicate approaches 1 (∼0.9; Figure 6D), indicating almost equal synthesis efficiency when the ratio with iodine oxidation was largely in disfavor of the gold slide (0.4).

With ITO substrates, the change in the oxidizer also brought about better hybridization results. Signals on the ITO slide treated with iodine and water were noticeably low at approximately 15% those of the glass copy (Figure 6). With CSO treatment, fluorescent signals on the ITO microarray reached close to 60% of the fluorescent signals on the glass duplicate, indicating a clear improvement in the synthesis efficiency. In all cases, scans display a uniform distribution of fluorescence with a low background signal intensity. Only the CSO-oxidized glass copy microarray from ITO synthesis is less satisfactory with regard to fluorescence homogeneity (Figure 6F). The signal/background ratio is markedly better for almost all CSO-oxidized DNA microarrays, therefore making it a method of choice for the synthesis on conductive substrates.

Despite clear improvements in the surface integrity and hybridization signals, DNA microarrays on gold or ITO substrates with CSO oxidation fail to yield fluorescence signal intensities as high as or higher than those of the glass copy. To understand whether this effect is still due to differences in the synthesis efficiency, we measured the amount of hybridized DNA on all types of surfaces and for all oxidation methods. To do so, the microarray synthesis was carried out over the entire available synthesis area. A complementary strand with a fluorescent tag was first hybridized to the array and then recovered in a minimal amount of buffer. The resulting sample was then quantified for Cy3 fluorescence and the DNA concentration compared to calibrated values (Figure S4 and Table S1). DNA recovery per slide approaches quantities expected for DNA libraries synthesized and cleaved from the surface.^53^ For glass surfaces, the concentration of recovered DNA can reach up to ∼6–7 pmol per array (Table 1), which is well within the range of available hydroxyl sites on glass microscope slides (rated at 0.1–1 pmol/mm^2^ or 1–10 pmol for a synthesis area of ∼1 cm^2^)^56^ and suggests that nearly all surface-bound DNA molecules are associated with a fluorescently labeled complementary strand. In general, the amount of recovered DNA from glass slides is noticeably lower when synthesis was carried out in combination with a gold substrate (4–5 pmol/array), which is in line with the recorded fluorescence intensities being on average lower than in the ITO/glass system (Figure 6F, compare the top and bottom rows). As expected, DNA hybridization onto the I_2_-oxidized gold substrate yields the lowest amount of recovered material at 1.6 pmol/array, suggesting a good correlation between signal intensities, visible deterioration, and synthesis efficiency. When replacing the oxidizer with CSO, not only was the recovered amount of hybridized DNA on gold well above that of the iodine-oxidized version (7.4 pmol/array), but it also surpassed the amount of DNA recovered from the glass duplicate (5.8 pmol/array). This result is in apparent contradiction with the 0.9 “dual array ratio” or ratio between hybridization signals recorded on the gold surface and its glass counterpart. This would suggest that the synthesis yield and/or efficiency on CSO-oxidized gold is greater than on glass but fluorescence scanning fails to present an accurate record of the Cy3 intensity on gold-coated slides. This observation would indicate fluorescence quenching on gold, which is a known phenomenon and occurs at distances relevant to this study,^57^ indicating that the physicochemical properties of gold have been unaffected by chemical synthesis, treatment, and subsequent handling.

**Table 1:** Quantification of Fluorescently Labeled Complementary DNA Hybridized to the Microarray Surface (in pmol/microarray) after a Microarray Wash-off^a^

oxidation	substrate	pmol/microarray
I_2_/H_2_O	ITO	4.28 ± 0.01
	glass copy	6.97 ± 0.26
	gold	1.62 ± 0.02
	glass copy	4.73 ± 0.38
CSO	ITO	5.14 ± 0.08
	glass copy	6.49 ± 0.12
	gold	7.43 ± 0.17
	glass copy	5.85 ± 0.10

a The concentration was measured in triplicate (*n* = 3) for all samples. Hybridization assays were performed under similar conditions for all substrates. Glass copy refers to the duplicate array synthesized on standard glass along with the conductive substrate

Hybridized DNA recovered from ITO slides shows that CSO oxidation indeed improves the synthesis yield relative to I_2_/H_2_O oxidation (5.1 vs 4.2 pmol/array, respectively), which is once again in line with signal intensities and with improved surface integrity. In the CSO case, the amount of recovered DNA from the ITO slide approaches 80% that from the glass slide (5 vs 6.5 pmol/array) when it is only ∼60% with iodine-mediated oxidation. Here, again, this ITO/glass DNA ratio is noticeably different from the ITO/glass fluorescence ratio (60 and 20%, respectively; see Figure 6D). This difference in the signal intensity that remains even after improving synthesis on ITO is likely due to fluorescence quenching. Fluorescence quenching on ITO slides has been reported for short (<10 nm) surface-to-dye distances and for film thickness/fluorophore combination relevant to this study.^58^

## Conclusions

The chemistry behind the synthesis of nucleic acid microarrays in situ can be as versatile as for solid-phase as it builds biopolymers using the same tools,^40,59−61^ but simultaneously, the technology faces the same challenges. In the context of our expanding repertoire of chemical modifications and expanding catalogue of synthesis substrates, we question whether in situ microarray photolithography can also cater to more sensitive synthesis conditions. In this study, we explore the use of CSO as a nonaqueous oxidizer instead of the standard aqueous iodine solution. We find that CSO-mediated oxidation works equally well for DNA microarray synthesis, yielding hybridization signals as high as for I_2_/H_2_O-mediated oxidation and only requires a stepwise reaction time of 20 s. Longer oligomers up to 100 nt can be synthesized in a higher purity with CSO too. Importantly, CSO oxidation dramatically improves the physicochemical properties of gold and ITO-coated slides, which are two electrically conductive substrates, relative to the DNA synthesis with iodine. The gold and ITO layers can be kept in pristine condition with no changes to their conductive properties, which translates into higher DNA synthesis yield, better hybridization efficiency, and brighter, cleaner fluorescence scans. These parameters will prove useful for the synthesis of DNA libraries on oxidizable surfaces for various non-fluorescence-based biosensing applications. Similarly, CSO oxidation on microarrays will help accommodate nucleoside and oligonucleotide analogues that are sensitive to the oxidizing conditions.

## Data Availability

Microarray scans for all experiments are available upon reasonable request.

## Supporting Information Available

Experimental details for off-array analysis (cleavage, wash-off, and gel and DNA quantitation), visual examination of the slides, final oxidation tests, and calibration for fluorescence measurements of washed-off DNA (PDF)
