# Supplementary material for: Nonaqueous Oxidation in DNA Microarray Synthesis Improves the Oligonucleotide Quality and Preserves Surface Integrity on Gold and Indium Tin Oxide Substrates
Source: Anal Chem. 2024 Jan 29;96(6):2378–86. doi: 10.1021/acs.analchem.3c04166 (PMC10867803; doi:10.1021/acs.analchem.3c04166)
Supplement: Supplementary file 1 — ac3c04166_si_001.pdf [file ac3c04166_si_001.pdf]

Supporting Information

Nonaqueous oxidation in DNA microarray synthesis improves oligonucleotide quality and preserves surface integrity on gold and indium tin oxide substrates

Erika Schaudy[1‖], Gisela Ibañez-Redín[1‖], Etkin Parlar[1], Mark M. Somoza[1,2,3] and Jory Lietard[1*]

[1]Institute of Inorganic Chemistry, University of Vienna, Josef-Holaubek-Platz 2, 1090 Vienna, Austria

[2]Leibniz-Institute for Food Systems Biology at the Technical University of Munich, Lise-Meitner-Straße 30, 85354 Freising, Germany

[3]Chair of Food Chemistry and Molecular Sensory Science, Technical University of Munich, Lise-Meitner-Straße 34, 85354 Freising, Germany

[‖]These authors contributed equally to this work

[*]jory.lietard@univie.ac.at

Table of Contents

Supporting Experimental Section 2

Library sequence, preparation, and analysis 4

Visual examination of DNA microarray synthesis on gold and ITO substrates 5

Final oxidation tests on gold-coated substrates 6

Fluorescence measurements of recovered DNA samples 6

Supporting Experimental Section

Off-array analysis

A single 97-nt long DNA oligonucleotide was synthesized over the entire available microarray surface and above a base-cleavable dT nucleotide immediately after synthesis of a dT_5 linker. The base-cleavable dT is a phosphoramidite with an ester functionality at the 3'-OH function (Thymidine-succinyl hexamide CED phosphoramidite, CLP-2244, ChemGenes) so as to render the array-synthesized oligonucleotide prone to base-mediated cleavage. Cleavage leaves behind a 3'-dT moiety, terminated with a 3'-OH function. To cleave oligonucleotides from the array, the deprotection step is instead performed in a dry 1:1 EDA/toluene solution for 2 h at r.t., followed by rinsing with 2 × 20 mL dry ACN, and finally applying 100 µl nuclease-free water over the synthesis area to recover the cleaved DNA. Dryness during deprotection is necessary to ensure that the DNA does not solubilize into water and escape into the deprotection solution, and that it remains electrostatically bound to the surface until addition of water. The cleaved DNA libraries were dried at 45 °C using a SpeedVac concentrator (Eppendorf). The sample was then rediluted by adding 10 µL of nuclease-free water. DNA concentration and A_{260} absorbance was measured on a Nanodrop spectrophotometer (Thermo Fisher). The DNA was then analyzed by denaturing polyacrylamide gel electrophoresis (7 M urea, 20% acrylamide), loading 100 ng of DNA along with 5 µL of 2× RNA loading dye (New England Biolabs), for a total of 10 µL loading volume. The mixture was heated at 70 °C for 10 minutes and immediately placed on ice for a few minutes before loading. An oligonucleotide size standard (IDT 51-05-15-02), diluted in loading dye to 50 ng each oligonucleotide, was loaded onto the same gel. Gel electrophoresis was carried out for 1 h at 30 V and 55 min at 40 V, the gel was subsequently immersed in a staining solution (SYBR Gold Nucleic Acid Gel Stain (1×) in 50 mL 1× TBE Buffer) and shaken at r.t. for 25 min. The stained gel was then scanned using a Fusion-FX7 fluorescence scanner, with exposure times varying from 5 to 10 seconds.

Hybridization wash-off experiments

DNA density was determined by adapting a previously described methodology[24]. In this procedure, a 25mer oligonucleotide sequence (5'-GTCATCATCATGAACCACCCTGGTC) was chemically synthesized across the entire synthesis area (1.1 cm × 1.4 cm). Subsequently, the DNA arrays were subjected to hybridization with the Cy3-labeled complementary oligonucleotide as described in the hybridization section. The hybridization solution in this case consisted of 150 µL 2× MES buffer, 13.3 µL acetylated BSA (10 mg/mL), 133.5 µL of 100 nM Cy3-labeled complementary oligonucleotide, and 3 µL of nuclease-free water. After drying, a hybridization chamber was positioned over the synthesis area. The arrays were incubated with a wash-off solution (40 mM KCl, 132 mM KOH) with continuous rotation for one hour at room temperature. The resulting solution was collected and diluted to a final volume of 600 µL using the same wash-off buffer. Calibration solutions containing the Cy3-labeled

complementary oligonucleotide at concentrations ranging from 1×10^{-11} to 5×10^{-8} M were similarly prepared in the wash-off buffer. Fluorescence measurements were conducted in triplicate for all samples using a multimode microplate reader (TECAN Spark). The density of hybridized DNA was measured for each sample using the calibrated fluorescence curve.

Library sequence, preparation, and analysis

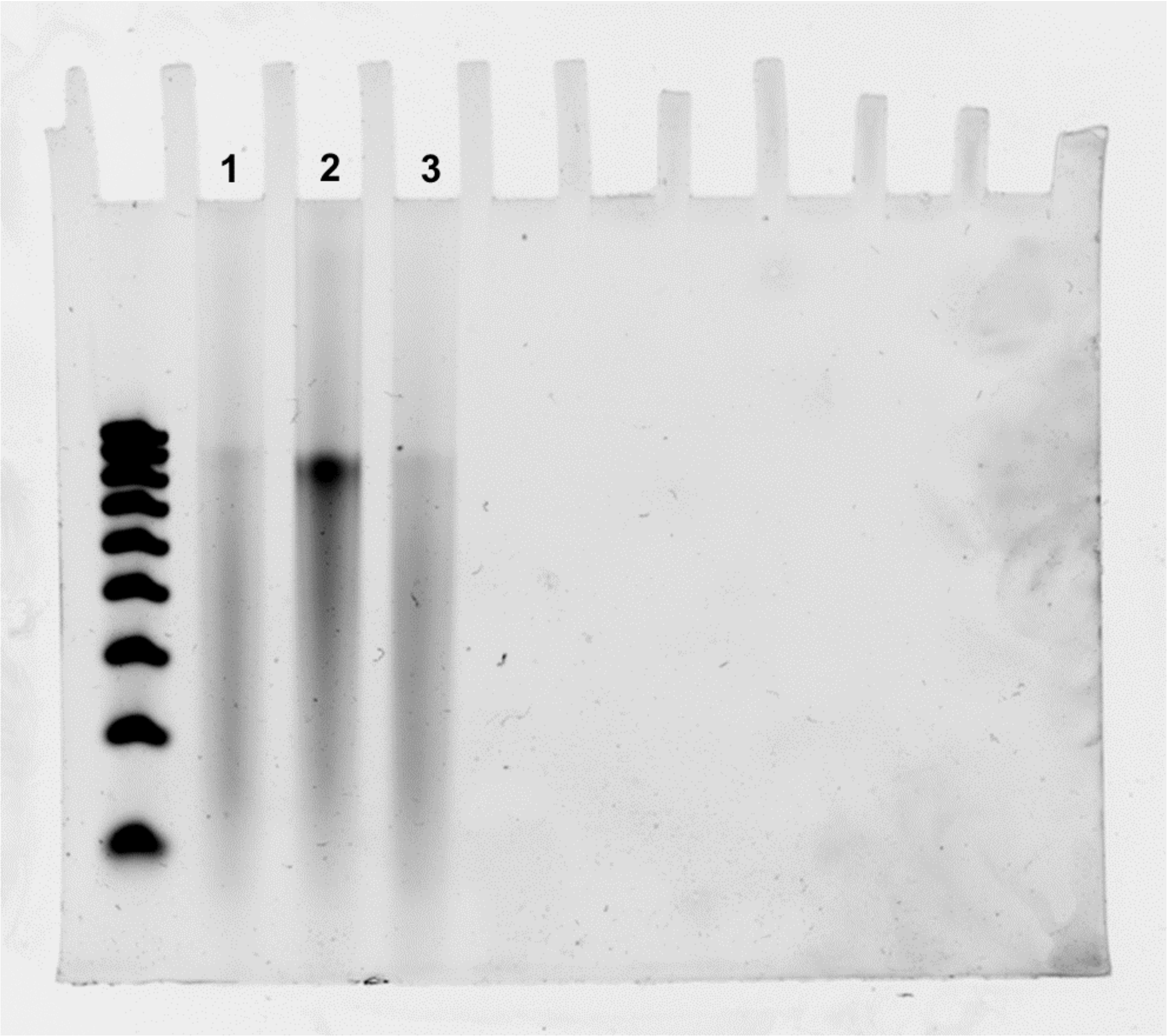

Figure S1. Denaturing 20% polyacrylamide gel electrophoresis of crude 97mers synthesized by MAS. The oxidation protocol was either I_2/H_2O (3 s stepwise, lane 1, or 30 s stepwise, lane 3) or 0.5 M CSO (20 s stepwise, lane 2). The oligonucleotides were synthesized with BzNPPOC DNA phosphoramidites, using ETT as activator and 6 J/cm^2 of radiant exposure. Amount loaded for each sample was 100 ng. Left: DNA ladder (20 – 100 nt).

The oligonucleotide synthesized as part of the cleavage and gel assays is a 97mer of the following sequence:

5´ACACTCTTTCCCTACACGACGCTCTTCCGATCTCGCAATGATGATATAGATCAGCCAGCAACAGATCGGAAGAG CACACGTCTGAACTCCAGTCACT

The final 3'-T corresponds to a base-cleavable dT phosphoramidite (ChemGenes, CLP-2244, diluted to 50 mM in ACN) that was coupled for 2 × 2 min over a standard dT_5 linker. The 5'-DMTr protecting group was removed by flushing the reaction chamber with a deblocking solution (3% trichloroacetic acid in dichloromethane) for 60 s before resuming light-mediated microarray synthesis of the remaining 96mer.

Visual examination of DNA microarray synthesis on gold and ITO substrates

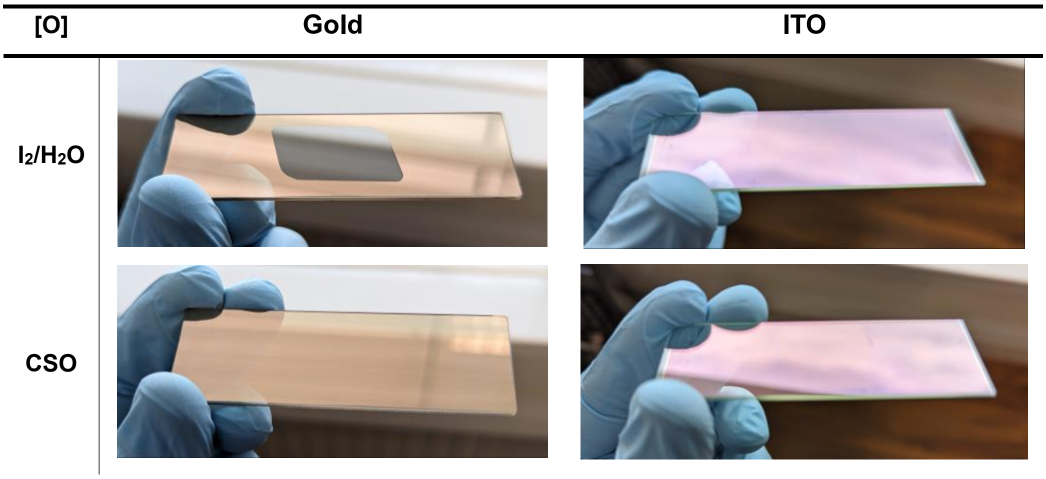

Figure S2. Visual examination of gold-coated and ITO-coated microscope slides after photolithographic DNA microarray synthesis with stepwise iodine- or CSO-mediated P(III) oxidation.

Final oxidation tests on gold-coated substrates

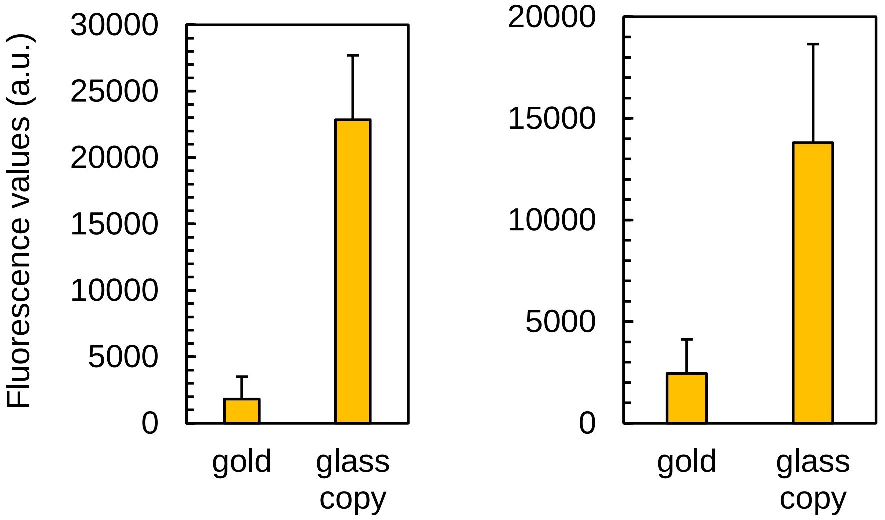

Figure S3. (left) Hybridization signals with a 25mer Cy3-labeled complementary strand onto DNA microarrays synthesized on gold and glass substrates with a final, off-cell oxidation step with 0.25 M H_2O_2 in ACN, for 10 min at r.t. (right) Similar setup but with a final, in-cell oxidation step with I_2/H_2O, for 3 s. The arrays were then washed in ACN and deprotected/hybridized as described in the experimental section. Error bars are S.D.

Fluorescence measurements of recovered DNA samples

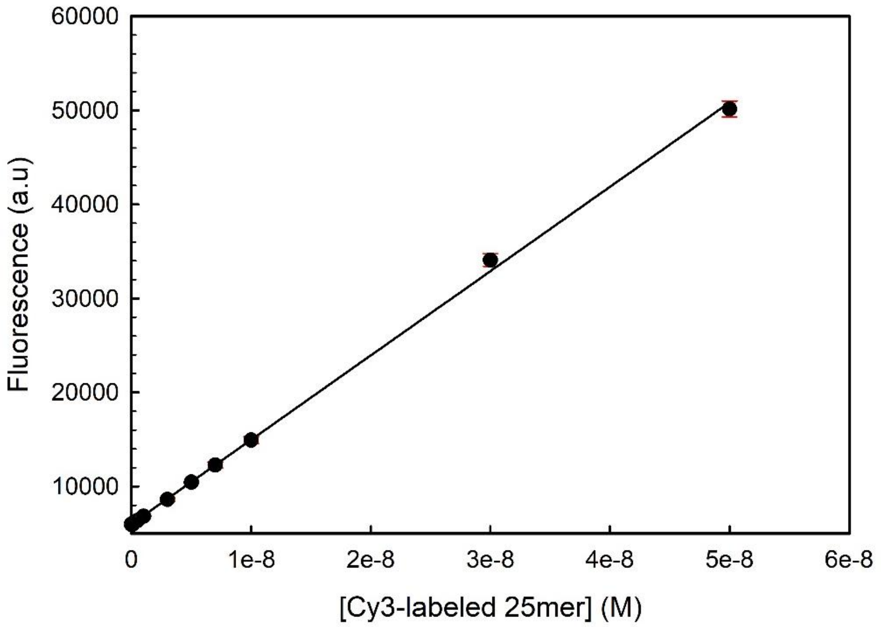

Figure S4. Calibration curve for the determination of DNA concentration using Cy3 fluorescence. A dilution series of an authentic, pure sample of Cy3-labeled 25mer complementary strand in the wash-off buffer (50 nM to 10 pM) was prepared and the fluorescence intensity of each sample recorded on a microplate reader. Each sample was measured in triplicate. Hybridized DNA recovered in the wash-off were then measured for fluorescence intensity and the values reported onto the calibration curve to obtain DNA concentration.

Table S1. Fluorescence measurements (in arbitrary units) of the recovered DNA sample in the wash-off buffer after hybridization to each microarray (ITO, gold or standard silanized glass, with I_2 or CSO-mediated oxidation). The DNA sample is a 25mer sequence complementary to the 25mer DNA synthesized over the entire synthesis area of the microarray. The hybridized strand is labeled at the 5′ end with Cy3. Each sample was measured in triplicates. * sample collected from the array was diluted to 900 µl before fluorescence measurements instead of 600 µl.

Slide/Oxidizer	Signal (a.u.)	Concentration (mol/L)	Mol	Density (mol/cm^2)
**ITO/I_2**	12406	7.14652E-09	4.28791E-12	2.78436E-12
**ITO/I_2**	12545	7.30152E-09	4.38091E-12	2.84475E-12
**ITO/I_2**	12232	6.9525E-09	4.1715E-12	2.70877E-12
**ITO/CSO**	13800	8.70094E-09	5.22056E-12	3.38998E-12
**ITO/CSO**	13674	8.56044E-09	5.13626E-12	3.33524E-12
**ITO/CSO**	13558	8.43109E-09	5.05865E-12	3.28484E-12
**Gold/I_2**	8458	2.7442E-09	1.64652E-12	1.06917E-12
**Gold/I_2**	8407	2.68733E-09	1.6124E-12	1.04701E-12
**Gold/I_2**	8413	2.69402E-09	1.61641E-12	1.04962E-12
**Gold/CSO**	17153	1.24398E-08	7.46387E-12	4.84667E-12
**Gold/CSO**	16821	1.20696E-08	7.24175E-12	4.70243E-12
**Gold/CSO**	17339	1.26472E-08	7.58831E-12	4.92748E-12
**Glass copy (ITO)/I_2**	16731	1.19692E-08	7.18153E-12	4.66333E-12
**Glass copy (ITO)/I_2**	15981	1.11329E-08	6.67975E-12	4.3375E-12
**Glass copy (ITO)/I_2**	16537	1.17529E-08	7.05174E-12	4.57905E-12
**Glass copy (ITO)/CSO**	15622	1.07326E-08	6.43956E-12	4.18153E-12
**Glass copy (ITO)/CSO**	15896	1.10381E-08	6.62288E-12	4.30057E-12
**Glass copy (ITO)/CSO**	15572	1.06769E-08	6.40611E-12	4.15981E-12
***Glass copy (gold)/I_2**	10838	5.39808E-09	4.85827E-12	3.15472E-12
***Glass copy (gold)/I_2**	11017	5.59768E-09	5.03791E-12	3.27137E-12
***Glass copy (gold)/I_2**	10280	4.77587E-09	4.29828E-12	2.79109E-12
**Glass copy (gold)/CSO**	14567	9.5562E-09	5.73372E-12	3.72319E-12
**Glass copy (gold)/CSO**	14833	9.85281E-09	5.91169E-12	3.83876E-12
**Glass copy (gold)/CSO**	14846	9.86731E-09	5.92038E-12	3.8444E-12
